# Playing a musical instrument and the risk of dementia among older adults: a systematic review and meta-analysis of prospective cohort studies

**DOI:** 10.1186/s12883-022-02902-z

**Published:** 2022-10-27

**Authors:** Ahmed Arafa, Masayuki Teramoto, Saori Maeda, Yukie Sakai, Saya Nosaka, Qi Gao, Haruna Kawachi, Rena Kashima, Chisa Matsumoto, Yoshihiro Kokubo

**Affiliations:** 1 Department of Preventive Cardiology, National Cerebral and Cardiovascular Center, 6-1, Kishibe- Shinmachi, 564-8565 Suita, Osaka Japan; 2 Department of Public Health, Faculty of Medicine, Beni-Suef University, Beni-Suef, Egypt; 3 Department of Epidemiology and Biostatistics, University of California, San Francisco, USA; 4 Department of Environmental Medicine and Population Sciences, Graduate School of Medicine, Osaka University, Suita, Japan; 5 Department of Cardiovascular Pathophysiology and Therapeutics, Graduate School of Medicine, Osaka University, Suita, Japan; 6 Department of Cardiology, Center for Health Surveillance and Preventive Medicine, Tokyo Medical University Hospital, Shinjuku, Japan

**Keywords:** Music, Dementia, Older adults, Systematic review, meta-analysis

## Abstract

**Background:**

Engaging in leisure activities was suggested to protect older adults from dementia. However, the association between playing a musical instrument and the risk of dementia is not well-established. This study aimed to investigate this association in older adults using a systematic review and meta-analysis of prospective cohort studies.

**Methods:**

Pooled hazard ratio (HR) and 95% confidence interval (CI) of having dementia for older adults playing a musical instrument were calculated using the random-effects model. We performed the *I*^2^ statistic to detect heterogeneity across studies and the test for funnel plot asymmetry to assess publication bias. The risk of bias assessment was conducted using the modified Newcastle–Ottawa Scale.

**Results:**

A total of three prospective cohort studies were found eligible: two from the U.S. and one from Japan. Playing a musical instrument, in the meta-analysis, was significantly associated with a decreased risk of dementia (HR = 0.64; 95% CI: 0.41, 0.98) among older adults. No signs of significant heterogeneity across studies (*I*^2^ = 23.3% and p-heterogeneity = 0.27) or publication bias (z= -1.3 and p-publication bias = 0.18) were identified.

**Conclusion:**

Playing a musical instrument was associated with a decreased risk of dementia among older adults. Older adults should be encouraged to engage in leisure activities, especially playing musical instruments.

**Supplementary Information:**

The online version contains supplementary material available at 10.1186/s12883-022-02902-z.

## Introduction

The incidence and prevalence of dementia have been increasing worldwide, especially in the aging societies in North America, Europe, and South-East Asia [1]. It is estimated that the global number of dementia patients will increase from 57.4 million in 2019 to 152.8 million in 2050 [2]. In addition to the physical and psychological pressures affecting patients, families, and healthcare providers, dementia has enormous financial burdens in terms of formal and informal medical and social care costs [3].

However, dementia is not inevitable, and some lifestyle risk factors such as avoiding smoking, refraining from excessive alcohol drinking, and practicing physical activity could reduce its risk [4, 5]. Among these lifestyle factors, leisure activities can increase cognitive reserve, enhance psychological well-being, and improve social relationships; therefore, they were suggested to protect from dementia and delay cognitive decline [6–18].

Playing a musical instrument is a common leisure activity that carries several favorable cognitive effects and improves mental health [19, 20]. However, the relationship between playing a musical instrument and the risk of dementia is not well-established. A few studies investigated the potential effects of musical activities on dementia; however, they were limited by the small number of dementia cases that did not allow for a conclusive association to be drawn and the cross-sectional design of some studies that did not allow for a temporal association to be detected [9–13]. A previous meta-analysis of two studies investigated the same association. However, it included a few dementia cases, and later research was published [21]. We, therefore, conducted an updated systematic review of prospective cohort studies investigating the association between playing a musical instrument and dementia risk before combing the results of eligible studies in a meta-analysis.

## Methods

### Literature search and study selection

**Fig. 1:**
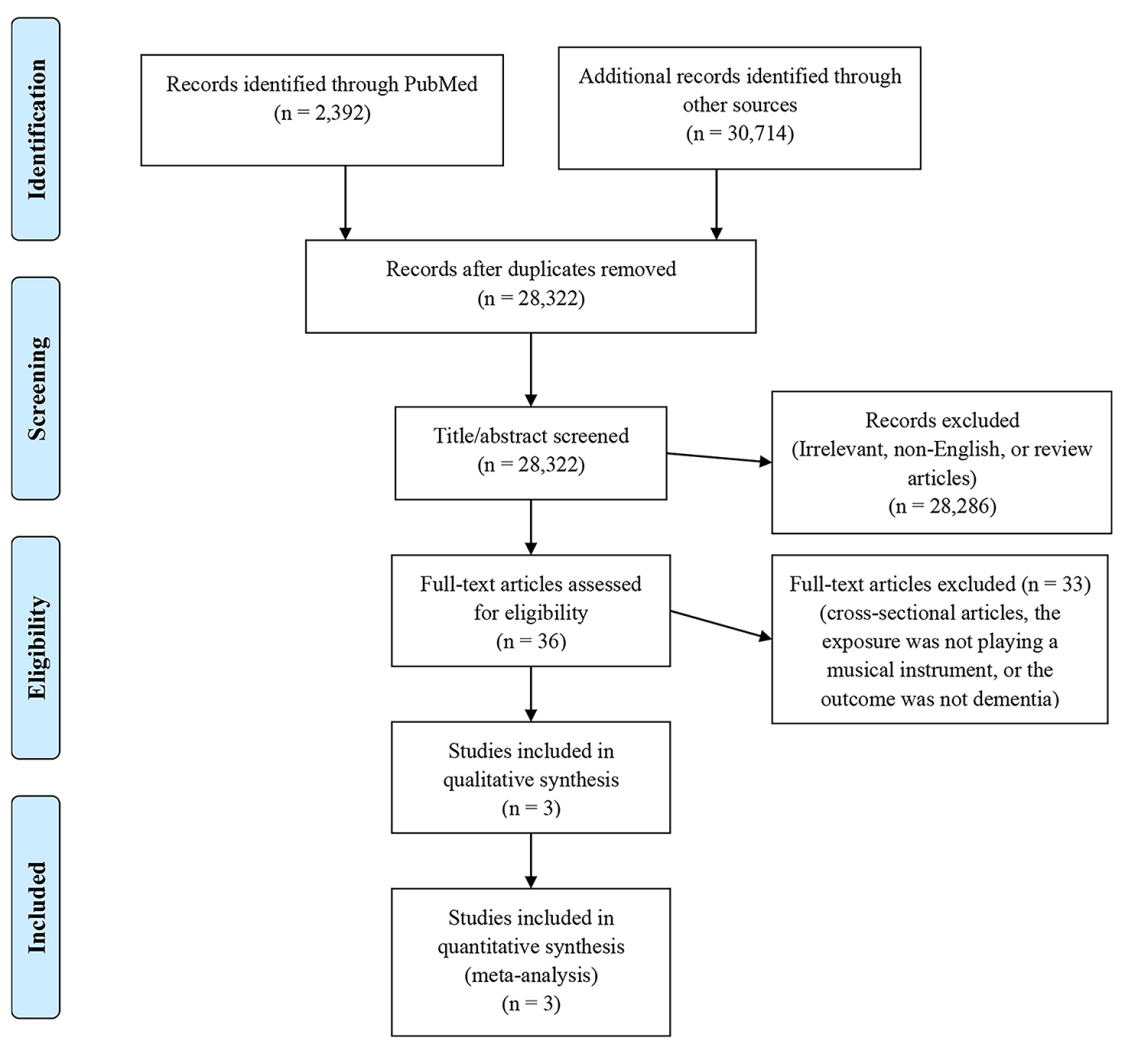
PRISMA flowchart of the selected articles to be included in the meta-analysis

First, we searched PubMed, Scopus, and Google Scholar for potential studies published in English before the 28th of February 2022 using the following terms: (musical instrument OR leisure activities) AND (dementia). A complete search strategy of PubMed was provided (Supplementary file 1). We also manually searched the reference lists of retrieved articles to obtain additional studies. We did not apply limitations regarding the year of publication, but no efforts were made to access unpublished data. We obtained 2,392 studies from PubMed, 2,614 from Scopus, and 28,100 from Google Scholar and the manual search of reference sections. After removing duplicates, we screened the titles and abstracts of 28,322 studies before removing non-English, irrelevant, and review articles, leaving 36 articles for full-text assessment (Fig. 1). Our eligibility criteria included: (1) the exposure was playing a musical instrument, (2) the outcome was dementia, and (3) the study had a prospective cohort design. Of note, our eligibility criteria, which were decided before conducting the systematic review, did not specifically define playing a musical instrument or whether studies investigating specific musical instruments would be only considered. We reported this meta-analysis according to the Preferred Reporting Items for Systematic Reviews and Meta-Analysis (PRISMA) checklist [22]. The following relevant information was extracted from the included studies by the first two authors: study name, publication year, place of study, characteristics of participants, assessment approaches of dementia and playing a musical instrument, follow-up years, number of dementia incident cases, and covariates included in the most adjusted regression models.

Later, we conducted another meta-analysis after including studies with cross-sectional design to investigate whether combining the results of cross-sectional studies with those from cohort studies would be different from confining the analysis to cohort studies only.

### Statistical analysis

We extracted the hazard ratios (HRs) with 95% confidence intervals (CIs) of dementia for playing a musical instrument in the most adjusted regression models before calculating the pooled HR (95% CI) of the three studies using the random-effects model [23]. We also conducted the *I*^2^ statistic to detect heterogeneity across studies [24] and the test for funnel plot asymmetry to assess publication bias [25]. The risk of bias was investigated using the modified Newcastle–Ottawa Scale in terms of studies’ selection, comparability, and outcome [26]. The first two authors assessed the risk of bias, with differences resolved by discussion. The R-3.2.0 statistical package (Metafor: Meta-Analysis Package for R) was used for analysis [27].

## Results

A shortlist of five studies was obtained [9–13]. We further excluded two cross-sectional studies [12, 13] and kept three prospective cohort studies for the main meta-analysis [9–11] (Fig. 1). The included studies used data from the Bronx Aging Study [9], the Monongahela Valley Independent Elders Survey (MoVIES) project [10], and the Japan Gerontological Evaluation Study (JAGES) [11]. Total participants, country, minimum age, percentage of women, follow-up period, and incidence of dementia in the included studies were as follows: Bronx: 469 participants, the U.S., 75 years, 64.0%, 5.1 years (median), and 26.4%, MoVIES: 942 participants, the U.S., 65 years, 66.5%, 6.1 years (mean), and 11.8%, and JAGES: 52,601 participants, Japan, 65 years, 53.9%, 5.8 years (median), and 11.0%. Methods of dementia diagnosis differed across the studies: Bronx: The Diagnostic and Statistical Manual of Mental Disorders, third edition or, after 1986, the revised third edition [28, 29], MoVIES: score ≥ one based on the Clinical Dementia Rating [30], and JAGES: level ≥ II according to the Standardized Dementia Scale of the Long-term Care Insurance System [31, 32]. Playing a musical instrument was assessed using an interview in the Bronx study (frequent versus rare) and a self-administered questionnaire in the MoVIES and JAGES studies (yes versus no). However, none of the included studies clarified the type of musical instrument or the duration of musical practice (Table 1).

**Table 1:** Summary of the studies included in the meta-analysis

Study ID	Population	Playing a musical instrument	Follow-up	Dementia	Adjusted variables
The Bronx Aging Study [9]	469 participants residing in one community in the US Age: 75–85 years Women: 64.0%	Interview (frequent or rare) Music players: 3.5%	5.1 years (median)	Incidence: 26.4% Diagnosed by the Diagnostic and Statistical Manual of Mental Disorders and the revised edition	Age, sex, educational level, medical illnesses, the Blessed Information–Memory–Concentration test, and participation in other leisure activities
MoVIES project [10]	942 participants from one rural area in the US Age: ≥65 years Women: 66.5%	Self-administered questionnaire (yes or no) Music players: 5.0%	6.1 years (mean)	Incidence: 11.8% Diagnosed by score ≥ 1 based on the Clinical Dementia Rating	Age, sex, education, depressive symptoms, physical exercise, functional impairment, self-reported health, medication use, and the recruitment status
JAGES [11]	52,601 participants representing 31 municipalities in 12 Japanese prefectures Age: ≥65 years Women: 53.9%	Self-administered questionnaire (yes or no musical activities at all) Music players: 2.5%	5.8 years (median)	Incidence: 11.0% Diagnosed by level ≥ II according to the Standardized Dementia Scale of the Long-term Care Insurance System	Age, sex, area, daily walking, mutual assistance, smoking, alcohol intake, marriage, education, annual income, engaging in other leisure cognitive activities, daily activities and meeting friends, body mass index, diabetes, hypertension, hyperlipidemia, stroke, hearing loss, and depression

**Fig. 2:**
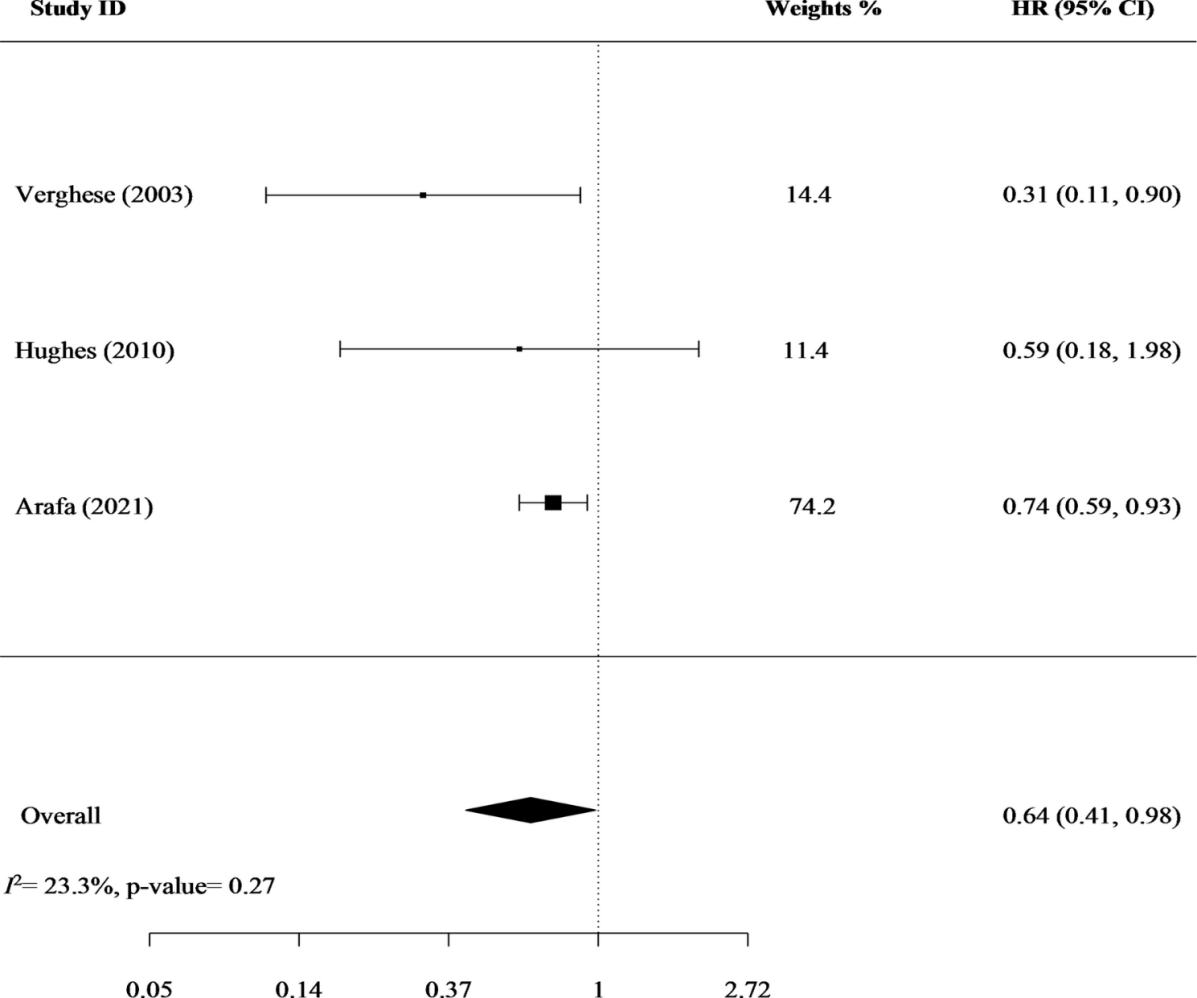
Playing a musical instrument and the risk of dementia

In the main meta-analysis that included the three cohort studies, playing a musical instrument was significantly associated with a decreased risk of dementia: pooled HR (95% CI) = 0.64 (0.41, 0.98), and the heterogeneity across studies was minimal (*I*^2^ = 23.3% and p-heterogeneity = 0.272) (Fig. 2). Also, no signs of publication bias were identified (z= -1.30 and p-publication bias = 0.182) (Supplementary file 2). Except for the short follow-ups, the included studies carried no major risks of bias (Supplementary file 3).

The two cross-sectional studies that were eliminated from the main meta-analysis investigated older adults ≥ 65 years from the Swedish HARMONY Co-twin Study (27 pairs where at least one twin was a musician) and the Japanese National Center for Geriatrics and Gerontology Study for Geriatric Syndromes Study (9,380 community-dwelling participants) [12, 13] (Supplementary file 4). The results did not change after pooling the odds ratios of the two cross-sectional studies [12, 13] with the HRs of the three prospective cohort studies [9–11] in one meta-analysis: Risk estimate (95% CI) = 0.68 (0.50, 0.92), *I*^2^ = 34.8%, and p-heterogeneity = 0.189 (Supplementary file 5).

## Discussion

This study provided epidemiological evidence from prospective cohort studies indicating an inverse association between playing a musical instrument and the risk of dementia among older adults. No signs of significant heterogeneity across studies or publication bias were detected, and the results did not change after including cross-sectional studies. Our findings came in line with a previous meta-analysis that suggested a protective effect of playing a musical instrument against dementia [21]. Yet, the previous meta-analysis included fewer dementia cases and did not consider pooling the results of prospective cohort studies with those from cross-sectional studies.

The biological explanation of the inverse association between playing a musical instrument and the risk of dementia is not well-understood. However, it could be attributed to the role of music in enriching cognitive reserve, enhancing executive functioning and working memory, stimulating brain plasticity to restore sensorimotor brain networks, reducing stress and depression, and developing social cohesion [33–40]. Cognitive reserve indicates individual differences in susceptibility to dementia-related brain changes. Older people with rich cognitive reserve, attained from leisure activities, can tolerate these changes and maintain cognitive functions [41]. Playing music can also reduce stress [37]. A previous meta-analysis showed that higher perceived stress, stressful events, and neuroticism were significantly associated with a higher risk of dementia [42]. Further, learning music and playing musical instruments in groups can be a chance for more social activities and widen the social network size. A 20-year prospective cohort study showed that engagement in social pursuits was related to delayed cognitive decline [43].

Engaging in other musical activities, such as karaoke, a type of interactive singing with music accompaniment and synchronized lyrics displayed on a video screen, was associated with a reduced risk of dementia in the JAGES [11]. Interestingly, in addition to preventing dementia, a recent systematic review and meta-analysis including 816 dementia patients from eight trials showed that music could be a novel therapy for treating dementia via improving cognitive functions, reducing depression and stress, and improving quality of life [44].

Our study included some limitations that should be considered. First, the outcome in the included studies was all-cause dementia. Risk factors for dementia could differ by dementia type [45]. Second, data on playing a musical instrument was self-reported suggesting reporting bias. However, one study concluded that the risk of cognitive impairment for self-reported playing a musical instrument was similar to the informant-reported risk; therefore, the possibility of reporting bias is not high [12]. Third, the duration of music playing was not collected; thus, a dose-response association could not be assessed. Fourth, the type of musical instrument and genre of practiced music were not assessed. Fifth, the short follow-up period might indicate a possibility of reverse causality. However, the JAGES results remained consistent after censoring the first three years of follow-up [11]. Sixth, we could not stratify our results by sex because only the JAGES calculated the sex-specified risk [11]. This point is specifically important because the protective effect of playing a musical instrument in the JAGES was more obvious among women than men [11]. Seventh, dementia is a complex disease with multiple intersecting genetic, clinical, and sociodemographic etiologies [46]; therefore, residual confounding cannot be excluded given the observational design of the included studies. Eighth, the meta-analysis was limited by the small number of included studies.

## Conclusion

In this meta-analysis, playing a musical instrument was associated with a reduced risk of dementia. Our findings support the evidence suggesting that engaging in cognitive leisure activities can reduce the risk of dementia. From a preventive perspective, older adults should be encouraged to engage in musical activities and other cognitive leisure activities. Given that leisure activities, including playing musical instruments, are modifiable lifestyle factors, the findings of our study highlight the importance of targeting leisure activities in risk prevention interventions and health education. Still, more prospective studies that consider the duration of playing music and the type of musical instruments are needed. Further studies to elaborate on the biological explanations of this association are warranted as well.

## Electronic supplementary material

Below is the link to the electronic supplementary material.

Additional File 1.

## Data Availability

All data generated or analyzed during this study are included in this published article.

## List of abbreviations

CI: Confidence Interval
HR: Hazard Ratio
JAGES: Japan Gerontological Evaluation Study
MoVIES: Monongahela Valley Independent Elders Survey
PRISMA: Preferred Reporting Items for Systematic Reviews and Meta-Analysis
