# Supplementary material for: Playing a musical instrument and the risk of dementia among older adults: a systematic review and meta-analysis of prospective cohort studies
Source: BMC Neurol. 2022 Oct 27;22:395. doi: 10.1186/s12883-022-02902-z (PMC9608922; doi:10.1186/s12883-022-02902-z)
Supplement: Supplementary file 1 — Additional File 1. [file 12883_2022_2902_MOESM1_ESM.docx]

**Supplementary file 1: PubMed search strategy**

(musical[All Fields] AND instrument[All Fields]) OR ("leisure activities"[MeSH Terms] OR ("leisure"[All Fields] AND "activities"[All Fields]) OR "leisure activities"[All Fields]) AND ("dementia"[MeSH Terms] OR "dementia"[All Fields])

**Supplementary file 2: Funnel plot for publication bias**

**Supplementary file 3: Risk of bias assessment using the Newcastle-Ottawa Quality Assessment Scale**

| Items | Verghese et al.,  (2003) | Hughes et al.,  (2010) | Arafa et al.,  (2021) |
| --- | --- | --- | --- |
| Representativeness of the exposed cohort | * | * | * |
| Ascertainment of exposure |  |  | * |
| Selection of the non-exposed cohort | * | * | * |
| Demonstration that the outcome of interest was not present at the start of the study | * | * | * |
| Comparability in terms of age, sex, and education | ** | ** | ** |
| Assessment of outcome | * | * | * |
| Follow-up was long enough for outcomes to occur |  |  |  |
| Adequacy of follow-up of cohorts | * | * | * |
| Overall (total number of asterisks) | 7 | 7 | 8 |

The possible overall scores could range between 0 and 9

**Supplementary file 4: Summary of the cross-sectional studies that investigated the association between playing a musical instrument and dementia**

| **Study ID** | **Population** | **Playing a musical instrument** | **Dementia** | **Adjusted variables** |
| --- | --- | --- | --- | --- |
| Balbag et al.,  (2014)  HARMONY  Twin Study | 27 pairs from Sweden where at least one twin was a musician  Age: ≥65 years | Questionnaire | Diagnostic and Statistical  Manual of Mental  Disorders, 4th Edition | -- |
| Kim et al.,  (2020)  NCGG-SGS | 9,380 community-dwelling older adults from Japan  Age: ≥65 years | Questionnaire | National Center for  Geriatrics and  Gerontology Functional  Assessment Tool | Age, sex, educational level, body mass index, employment status, medication use, current smoking habit, current alcohol use, grip strength, pain, and Geriatric Depression Scale score |

**Supplementary 5: Playing a musical instrument and the risk of dementia (prospective and cross-sectional studies)**
